# Supplementary material for: Reconciling Mining with the Conservation of Cave Biodiversity: A Quantitative Baseline to Help Establish Conservation Priorities
Source: PLoS One. 2016 Dec 20;11(12):e0168348. doi: 10.1371/journal.pone.0168348 (PMC5173368; doi:10.1371/journal.pone.0168348)
Supplement: S1 Dataset — (ZIP) [file pone.0168348.s002.zip › Taxa/Serra Norte/SN_2013/N4WS/Lista N4WS-70.pdf]

CAVIDADE N4WS-0070							
**Classe**	**Ordem**	**Fam/Outros**	**Gên/Outros**	**Espécie**	**S-U**	**U-S**	Zon.
Annelida	Oligochaeta			sp.	2		P
Arachnida	Acari	Ixodidae	*Amblyomma*	sp.1	X		P
Arachnida	Acari	Laelapidae	*Cosmolaelaps*	sp.1	X		P
Arachnida	Acari	Trombiculidae		sp.3	X		P
Arachnida	Amblypygi	Charinidae	*Charinus*	jovem	1		P
Arachnida	Amblypygi	Phrynidae	*Heterophrynus*	*longicornis*	6	1	P
Arachnida	Araneae	Barychelidae		jovem		1	P
Arachnida	Araneae	Ctenidae		jovem	1		P
Arachnida	Araneae	Dipluridae		jovem	1		P
Arachnida	Araneae	Drymusidae	*Drymusa*	*spelunca*	X	X	P
Arachnida	Araneae	Ochyroceratidae	*Speocera*	sp.1	X	X	P
Arachnida	Araneae	Pholcidae	*Mesabolivar*	*eberhard*		X	P
Arachnida	Araneae	Prodidomidae		jovem		X	P
Arachnida	Araneae	Symphytognathidae	*Anapistula*	sp.1	X		P
Arachnida	Araneae	Theraphosidae		jovem	2	2	P
Arachnida	Araneae	Theridiidae	*Theridion*	sp.2		X	P
Arachnida	Araneae	Theridiosomatidae		jovem	X		P
Arachnida	Opiliones	Sclerosomatidae		jovem		X	P
Arachnida	Opiliones	Stygnidae		jovem		1	P
Arachnida	Pseudoscorpiones	Chernetidae	*Spelaeochernes*	sp.1	X	X	P
Chilopoda	Geophilomorpha	Geophilidae		sp.1	1		P
Entognatha	Collembola	Paronellidae		sp.1	X	X	P
Entognatha	Diplura	Campodeidae		sp.1	X	X	P
Entognatha	Diplura	Projapygidae		sp.1		X	P
Insecta	Coleoptera	Staphylinidae		sp.35		X	P
Insecta	Coleoptera	Staphylinidae		sp.75	X		P
Insecta	Coleoptera	Tenebrionidae		jovem	X		P
Insecta	Diptera	Ceratopogonidae		sp.	X		P
Insecta	Diptera	Psychodidae	*Sciopemyia*	*sordellii*	X	X	P
Insecta	Heteroptera	Reduviidae	Emesinae	sp.3	1	1	P
Insecta	Heteroptera	Reduviidae	Reduviinae	jovem		1	P
Insecta	Hymenoptera	Formicidae	*Camponotus*	sp.1	X	X	P
Insecta	Hymenoptera	Formicidae	*Octostruma*	sp.1	X	X	P
Insecta	Hymenoptera	Formicidae	*Pheidole*	sp.2	X		P
Insecta	Hymenoptera	Formicidae	*Solenopsis*	sp.2		X	P
Insecta	Lepidoptera	Noctuoidea		sp.2	X		P
Insecta	Orthoptera	Phalangopsidae	*Paraclodes*	sp.	4		E
Insecta	Orthoptera	Phalangopsidae	*Phalangopsis*	sp.	23	10	P
Symphyla		Scutigerellidae	*Hanseniella*	sp.1	X		P
Amphibia	Anura	Strabomantidae	*Pristimantis*	cf. *fenestratus*	2		P
Mammalia	Chiroptera	Emballonuridae	*Peropteryx*	sp.		1	P
